# Rhabdomyolysis and Dengue Fever: A Case Report and Literature Review

**DOI:** 10.1155/2013/101058

**Published:** 2013-01-08

**Authors:** Tanya Sargeant, Tricia Harris, Rohan Wilks, Sydney Barned, Karen Galloway-Blake, Trevor Ferguson

**Affiliations:** ^1^Department of Medicine, The University of the West Indies, Mona, Kingston 7, Jamaica; ^2^Epidemiology Research Unit, Tropical Medicine Research Institute, University of the West Indies, Mona, Kingston 7, Jamaica

## Abstract

The medical literature contains only a few reports of rhabdomyolysis occurring in patients with dengue fever. We report the case of a 25-year-old Jamaican man who was admitted to a private hospital four days after the onset of an acute febrile illness with fever, myalgia, and generalized weakness. Dengue fever was confirmed with a positive test for the dengue antigen, nonstructural protein 1. He remained well and was discharged on day 6 of his illness. On day 8, he started to pass red urine and was subsequently admitted to the University Hospital of the West Indies. On admission he was found to have myoglobinuria and an elevated creatine phosphokinase (CPK) of 325,600 U/L, leading to a diagnosis of rhabdomyolysis. Dengue IgM was positive. He was treated with aggressive hydration and had close monitoring of his urine output, creatinine, and CPK levels. His hospital course was uneventful without the development of acute renal failure and he was discharged after 14 days in hospital, with a CPK level of 2463 U/L. This case highlights that severe rhabdomyolysis may occur in patients with dengue fever and that early and aggressive treatment may prevent severe complications such as acute renal failure and death.

## 1. Introduction

Rhabdomyolysis is characterized by the rapid breakdown of skeletal muscle with leakage of muscle cell contents into the circulation [1, 2]. These contents include electrolytes, myoglobin and other sarcoplasmic proteins, such as creatine kinase, lactate dehydrogenase, alanine aminotransferase, and aspartate aminotransferase [1]. The resulting myonecrosis presents clinically as limb weakness, myalgia and commonly, gross pigmenturia without haematuria [1, 2]. Acute renal failure is a common complication of rhabdomyolysis and is due to the toxic effects of filtering excessive quantities of myoglobin in the setting of hypovolaemia [3]. The causes of rhabdomyolysis are protean, with acute viral infections such as influenza, HIV, coxsackievirus, and cytomegalovirus recognized as common causes [1, 4]. Although the dengue virus shares several features with other viruses known to cause myopathies, dengue fever is not listed as a cause of rhabdomyolysis in major textbooks and review articles [4]. Over the last decade, a number of case reports of patients with dengue and rhabdomyolysis have been published in the literature [4–8]. We now report another case of rhabdomyolysis associated with dengue fever and present a literature review with a discussion of the clinical implications.

## 2. Case Presentation

A 25-year-old man with no known chronic illnesses presented to the University Hospital of the West Indies (UHWI) in Kingston, Jamaica, with a history of back pain and fever beginning eight days prior to his presentation. The fever lasted for two days and settled with the use of paracetamol. He then started to experience severe arthralgia, myalgia, and generalized muscle weakness which lasted for the first few days of his illness. He was seen by his primary care physician three days after the onset of symptoms and was admitted to a private hospital with a diagnosis of dengue fever. He reported that his platelet count at that time was 44,000/*μ*L but remained asymptomatic, with no signs of bleeding, and was therefore discharged with a plan for followup as an outpatient. Two days later he noticed dark-coloured, then red urine and therefore returned to the private hospital and was subsequently referred to the UHWI for further management.

At the time of presentation, there were no reports of mucosal bleeding, skin haemorrhages, or haematochezia. There was no history of retro-orbital pain, neck stiffness, or photophobia. There were no other urinary symptoms and no history of nausea or vomiting. Of note, he gave no history of exposure to rats and he had not travelled recently. However, he did recall a neighbour having a diagnosis of dengue fever 1-2 weeks prior to the onset of his symptoms. 

On physical examination, he had normal vital signs with a temperature of 36.3°C. His cardiovascular, respiratory, abdominal, musculoskeletal, and central nervous systems were all normal. Examination of the skin did not reveal any petechiae, purpurae, ecchymoses, or rash. There were no wet purpurae in his mouth and fundoscopy was negative for retinal haemorrhages. A tourniquet test was performed and was negative. Urinalysis revealed a pH of 7.5 and tested positive for blood with no other abnormalities. 

**Figure 1:**
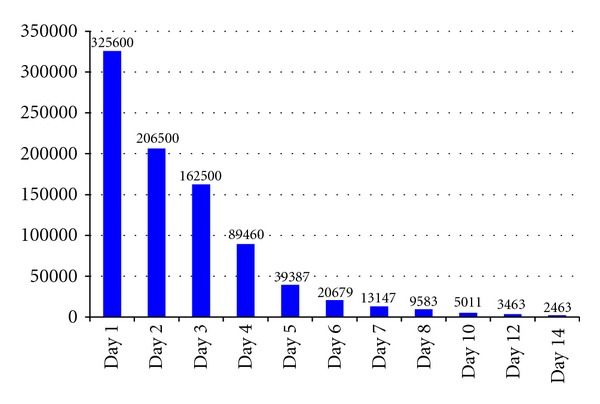
Trends in creatine phosphokinase (CPK) levels for case patient while in hospital.

Investigations from the private hospital revealed an initial leukopaenia, neutropaenia, and thrombocytopaenia, verified by peripheral blood film. By the time of his presentation to the UHWI, his white blood cell count had normalized but the platelet count remained low at 49,000/*μ*L. However, manual review of the peripheral blood film revealed a higher count of 100,000/*μ*L. His initial urea, creatinine, electrolytes, and coagulation studies were normal and remained normal for the duration of his admission. Creatinine level was 63 *μ*mol/L on admission and 82 *μ*mol/L at discharge. Admission aspartate transaminase was 1841 U/L (normal 7–32 U/L), *γ*-glutamyl transferase was 159 U/L (normal 10–70 U/L), and lactate dehydrogenase was 5740 U/L (normal 105–200 U/L). These values declined steadily during the admission and eventually normalized by the time of discharge. His initial creatine phosphokinase (CPK) was 325,600 U/L (normal for a male 40–240 U/L). With early and aggressive treatment, this value decreased by about 30–50% every day until it was 2,463 U/L at the time of discharge (see Figure 1). Urine microscopy was negative for red blood cells, casts, or other abnormal urinary sediments, but urinary myoglobin was positive. Tests for HIV, hepatitis B and C, and leptospirosis were all negative. Laboratory tests for the dengue virus on day four of his illness revealed a positive test for the dengue virus antigen, non-structural protein 1 (NS1), and negative immunoglobulin M (IgM). Dengue IgM became positive on day eight and remained positive on day 14. 

In-hospital management included aggressive hydration with intravenous and oral fluids, strict input/output charting, daily urinalyses to monitor pH, and close monitoring of the urea, creatinine, electrolytes, CPK, and platelet count. Target urine output was 200 mL/hour as recommended by Bosch and colleagues [1]. Urinary pH remained above 6.0 throughout and therefore alkalinization of the urine with sodium bicarbonate was not instituted. The patient's hospital course was uncomplicated, he remained clinically well and was discharged with instructions to return initially for review on the ward and then for followup in the out-patient clinic.

## 3. Discussion

This patient fulfils the criteria for a confirmed case of dengue fever as defined by the World Health Organization (WHO) [9] having presented with an acute febrile illness associated with headache, myalgia, arthralgia and leukopaenia and occurring at the same location and time as another confirmed case of dengue fever. It was verified by a positive IgM antibody test on the late acute and convalescent serum specimens and there was also demonstration of dengue virus antigen (NS1) in his serum. His was also a case of rhabdomyolysis with the typical triad of generalized weakness, myalgia, and dark urine/myoglobinuria associated with a CPK that was more than five times the upper limit of normal [2]. Although there are case reports of rhabdomyolysis associated with dengue fever, major textbooks do not mention the dengue virus as a possible cause of rhabdomyolysis [4, 7]. We found only one review article on atypical manifestations of dengue fever which includes rhabdomyolysis as a possible complication [10]. 

A review of the published literature using the PubMed database identified five case reports of rhabdomyolysis associated with dengue fever [4–8]. The findings of the six patients in these case reports and the present case are summarized in Table 1. In each case, there was confirmation of both dengue fever and rhabdomyolysis. Of note six of the seven cases were males, with ages ranging from 25 to 66 years; the lone female was 28 years old. CPK levels ranged from 5,000 to 325,600 U/L. Two of the seven patients died (29%) while four of them developed acute renal failure (57%) and two patients developed respiratory failure (29%). These clinical findings suggest that rhabdomyolysis in patients with dengue carries high morbidity and mortality rates when compared to the 8% overall mortality and 13–50% incidence of acute renal failure for all patients with rhabdomyolysis [1, 2]. In the cases presented, mortality was associated with the presence of acute renal failure and/or multiorgan failure. 

The mechanisms involved in the development of rhabdomyolysis in patients with dengue fever are unknown [4]. However, rhabdomyolysis is reported to occur in a number of viral infections, such as influenza A and B, coxsackievirus, Epstein-Barr virus, and HIV [1]. Davis and Bourke suggest that since the dengue virus shares several features with these other viruses known to cause severe myositis, it is not surprising that dengue could also cause rhabdomyolysis [4]. They further suggest that the most likely cause may be due to myotoxic cytokines, particularly tumour necrosis factor (TNF) and interferon alpha (IFN-*α*) released in response to a viral infection [4]. Muscle biopsy specimens from patients with acute viral myositis have revealed a range of findings, from a mild lymphocytic infiltrate to foci of severe myonecrosis but direct invasion of muscle by the virus has not been consistently demonstrated [4]. 

Acute kidney injury associated with myoglobinuria is the most serious complication of both traumatic and nontraumatic rhabdomyolysis and may be life-threatening. As seen in the cases reviewed, acute renal failure is a frequent complication in patients with dengue and rhabdomyolysis. The exact mechanisms by which rhabdomyolysis impairs renal function are unclear, but experimental evidence suggests that intrarenal vasoconstriction, direct and ischaemic tubular injury, and tubular obstruction all play a role [1]. Myoglobin becomes concentrated along the renal tubules, where it precipitates when it interacts with the Tamm-Horsfall protein, particularly in the presence of acidic urine. Myoglobin seems to have no marked nephrotoxic effect in the tubules unless the urine is acidic, hence the common practice of urinary alkalinization as part of supportive treatment measures. 

There is no specific threshold value of serum CPK above which the risk of acute kidney injury is markedly increased, but the risk is usually low when CPK levels at admission are less than 15,000–20,000 U/L. Acute kidney injury has been reported with CPK values as low as 5000 U/L, but this usually occurs with coexisting conditions such as sepsis, dehydration, and acidosis [1]. In the published case reports of dengue and rhabdomyolysis, CPK levels ranged from a low of 5,000 U/L to a high of 156,000 U/L. Our patient had an even higher CPK level of 325,600 U/L but did not develop acute renal failure. 

## 4. Conclusion

Rhabdomyolysis should be recognized as a possible complication of dengue fever and should be reflected as such in medical textbooks especially in light of the possibility of acute renal failure and high mortality. Although causation has not been unequivocally established, supporting evidence for a causal association is the fact that rhabdomyolysis occurs commonly with influenza and other viruses with which the dengue virus shares many similarities. 

Clinicians should note that the presentation of rhabdomyolysis can be subtle but its complications, in particular, acute kidney injury and multiorgan failure, can be devastating. These adverse effects are preventable with early recognition and institution of the appropriate management. We agree with Davis and Bourke that all patients with dengue fever should have a urinalysis done and that those who test positive for blood should have urine microscopy and a CPK test in order to determine if the patient may have rhabdomyolysis. This approach could be potentially life-saving. 

## Figures and Tables

**Table 1:** Summary of clinical findings from case reports of dengue fever and rhabdomyolysis.

Authors and publication year	Country, age, sex	Clinical findings	CPK level (Admission/peak) (U/L)	Results of tests for dengue	Renal function/acute renal failure	Outcome
Gunasekera et al. 2000 [6]	Sri Lanka 28 years, Female	Fever, myalgia, proximal muscle weakness, dark urine, dyspnea Acute renal failure, hypotension	>5000	Dengue IgM and IgG were positive by immunochromatography test Dengue antibody titre for D2 was 2560 (normal <20)	Developed acute renal failure—serum creatinine peaked at 780 *μ*mol/L Abdominal ultrasound showed normal kidneys	Complete and uneventful recovery after supportive treatment including mannitol and bicarbonate

Davis and Bourke 2004 [4]	Darwin, Australia 33 years, Male	Fever, retro-orbital pain, malaise, macular rash, dark red-brown urine	51,555	Dengue virus IgM negative on day 1 but positive on day 10 RT-PCR for serum dengue virus RNA on day 1 of hospitalization was positive for D2	Did not develop renal failure—serum creatinine levels were normal (actual values were not reported)	Uncomplicated course with full recovery

Davis and Bourke 2004 [4]	Darwin, Australia 33 years, Male	Admitted to hospital with suspected dengue haemorrhagic fever and acute renal failure	17,548	Blood RT-PCR was positive for D2	Developed acute renal failure—actual creatinine values were not reported	Developed multiple organ dysfunction Died on day 2 of hospitalization

Lim and Goh 2005 [8]	Singapore 27 years, Male	Four-day history of fever, nausea, and myalgia; discharged and readmitted after presenting with persistent myalgia and dark red urine	58,961	Dengue virus IgM and IgG titres were negative on day 1 of hospitalization Dengue IgM became positive on day 5	Did not develop acute renal failure—serum creatinine levels reported as normal (actual values were not given)	Uneventful hospital stay with discharge on day 7 of second admission after treatment with intravenous hydration

Karakus et al. 2007 [7]	The Netherlands 66 years, Male	Fatigue, muscle, bone, and joint pain, tea coloured urine; confirmed myoglobinuria	156,900	Dengue IgM and IgG positive on day 9	Developed acute renal failure—serum creatinine 138 *μ*mol/L on day 1 and 315 *μ*mol/L on day 5	Developed respiratory failure, septic shock and multiorgan failure; admitted to intensive care unit; Died on day 47

Acharya et al. 2010 [5]	India 44 years, Male	Four-day history of fever and myalgia On examination, he was febrile and had conjunctival congestion and diffuse muscle tenderness Urine positive for myoglobin	29,000	Dengue IgM ELISA was strongly positive	Developed acute renal failure—serum creatinine 2.6 mg/dL	Developed quadriparesis and respiratory failure Required ventilator support for respiratory failure

Present case (Sargeant et al. 2012)	Jamaica 25 years old, Male	Four-day history of fever, myalgia and generalized weakness. Started to pass red urine on day 8; myoglobinuria confirmed on urine testing	325,600	Dengue IgM negative on day 4 but positive on day 8 Dengue antigen (NS1) positive on day 4	Did not develop renal failure—serum creatinine was 63 *μ*mol/L on admission and 82 *μ*mol/L at discharge	Full recovery with supportive care

CPK: creatine phosphokinase; IgM: immunoglobulin M; IgG: immunoglobulin G, D2: dengue virus type 2, NS1: nonstructural protein 1.
